# Oral Fibrosis and Oral Cancer: From Molecular Targets to Therapeutics

**DOI:** 10.3390/ijms23116110

**Published:** 2022-05-30

**Authors:** Pei-Ling Hsieh, Cheng-Chia Yu

**Affiliations:** 1 Department of Anatomy, School of Medicine, China Medical University, Taichung 404333, Taiwan; plhsieh@mail.cmu.edu.tw; 2 School of Dentistry, Chung Shan Medical University, Taichung 40201, Taiwan; 3 Department of Dentistry, Chung Shan Medical University Hospital, Taichung 40201, Taiwan; 4 Institute of Oral Sciences, Chung Shan Medical University, Taichung 40201, Taiwan

Oral submucous fibrosis (OSF) belongs to a group of potentially malignant disorders that are characterized by the progressive fibrosis of the lining mucosa as well as an increasing loss of tissue mobility. Patients with OSF often experience discomfort caused by ulceration, xerostomia, or a burning sensation. A number of epidemiological factors have been implicated in the central mechanisms influencing the metabolism of extracellular matrix (ECM) molecules and the development of OSF. Among those, the habit of areca nut chewing has been studied extensively, and other factors, such as genetic mutations, human papilloma virus (HPV) infection, and nutritional deficiencies, are linked to the etiology of excessive ECM accumulation as well. It is well known that repetitive inflammation due to areca nut irritation, the impairment of collagen homeostasis, and the alteration of epithelial mesenchymal transition (EMT) molecules contribute to the progression of OSF.

One of the most dominant cell types in the regulation of fibrogenesis is myofibroblasts, which are spindle-shaped cells that express α-smooth muscle actin (α-SMA) and are responsible for remodeling ECM components. Myofibroblasts are activated fibroblasts in of the context of wound healing under physiological conditions; however, the persistent activation of myofibroblasts results in the accumulation of collagen and subsequent tissue or organ fibrosis. It has long been recognized that tumors are a type of non-healing wound [1], and various similarities between cancer-associated fibroblasts (CAFs) and fibrosis-associated fibroblasts (FAFs) have been reported at the cellular level. Moreover, numerous studies have suggested that myofibroblasts affect cancer metabolism by recruiting immune cells and regulating tumor immunity [2]. In the recent years, several clinical interference studies determining the action of CAFs have been conducted to explore potential patient benefits [3]. Hence, targeting myofibroblasts may be a feasible approach to alleviate the malignant progression of OSF into oral cancer.

Accumulating evidence has suggested that non-coding RNAs are involved in the regulation of myofibroblast activation through transcriptional or post-transcriptional modulation. Currently, several types of non-coding RNAs have been revealed, such as short (e.g., microRNA; ~22 nucleotides), long (>200 nucleotides), or circular RNAs. Various microRNAs and long non-coding RNAs have been demonstrated to mediate key molecules/pathways (e.g., EMT regulators or TGFβ signaling) in the transdifferentiation of myofibroblasts from normal buccal mucosal fibroblasts. Additionally, there have been efforts elucidate the interactions among long non-coding RNA, microRNA, and their target genes during myofibroblast activation. Several studies have revealed that some long non-coding RNAs act as competing endogenous RNAs to titrate the effect of certain microRNAs through the microRNA response elements. Additionally, multiple methods to reverse the aberrant expression of these non-coding RNAs, such as herbal medicine [4] or extracellular vesicles [5], have gained promising results in terms of alleviating oral fibrogenesis. Nevertheless, how to translate the optimism of in vitro results into real clinical benefits requires additional effort.

It is well-recognized that the etiology of oral cancer appears to be multifactorial and that has is genetic, epigenetic, and habitual causes (areca nut/cigarette/alcohol use). A mutation in p53 is one of the most investigated genes, and various studies have focused on advances in genomic research. Non-coding RNAs also play critical roles in oral carcinogenesis with a background in OSF [6]. Without a doubt, they are key players in orchestrating oncogenicity, cancer stemness, drug resistance, and the metastasis of oral cancer [7,8]. Aside from regulating EMT-associated factors, non-coding RNAs mediate the abovementioned events through various aspects, such as the modulation of the genes associated with cellular metabolism, TGFβ, Wnt, or Akt signaling pathways [9,10]. Furthermore, non-coding RNAs are critical to tumor immune escape [11] and participate in the success of using immune-checkpoint inhibitor therapy [12]. Similarly, non-coding RNAs can serve as therapeutic targets of oral cancer via numerous means, such as via exosomes [13] and nanoparticles [14]. They also have been suggested to predict the prognosis of patients with oral cancer [15]. The clinical implication of these non-coding RNAs requires further validation in the future.
